# Modulation by Estradiol of L-Dopa-Induced Dyskinesia in a Rat Model of Post-Menopausal Hemiparkinsonism

**DOI:** 10.3390/life12050640

**Published:** 2022-04-26

**Authors:** Kaja Kolmančič, Marko Živin, Maja Zorović

**Affiliations:** 1 Clinical Department of Nuclear Medicine, Ljubljana University Medical Centre, 1000 Ljubljana, Slovenia; kaja.kolmancic@kclj.si; 2 Brain Research Laboratory, Institute of Pathophysiology, Medical Faculty, University of Ljubljana, 1000 Ljubljana, Slovenia; marko.zivin@mf.uni-lj.si

**Keywords:** Parkinson’s disease, ovariectomy, 6-OHDA, 17-β-estradiol (E2), L-dopa-induced dyskinesia (LID), abnormal involuntary movements (AIMs), contralateral rotation, forepaw adjusted step (FAS) test, tyrosine hydroxylase (TH), ΔFosB

## Abstract

Treatment with levodopa (L-dopa) in Parkinson’s disease (PD) leads to involuntary movements termed L-dopa-induced dyskinesia (LID). There are contradictory data about the influence of hormone therapy in female PD patients with LID and of 17-β-estradiol (E2) on animal correlates of LID-abnormal involuntary movements (AIMs). Our aim was to characterize the influence of E2 on motor impairment and AIMs in ovariectomized 6-hydroxydopamine (6-OHDA) rat model of PD. Half of the rats received empty and the other half implants filled with E2. Following the 6-OHDA surgery, the rats received daily treatment with either L-dopa or saline for 16 days. They were assessed for AIMs, contralateral rotations, and FAS. In the L-dopa-treated rats, E2 intensified and prolonged AIMs and contralateral rotations. On the other hand, it had no effect on motor impairment. Postmortem tyrosine hydroxylase immunostaining revealed an almost complete unilateral lesion of nigrostriatal dopaminergic neurons. E2 partially prevented the upregulation of striatal ΔFosB caused by dopamine depletion. L-dopa potentiated the upregulation of ΔFosB within the dopamine-depleted striatum and this effect was further enhanced by E2. We speculate that the potentiating effects of E2 on AIMs and on contralateral rotations could be explained by the molecular adaptations within the striatal medium spiny neurons of the direct and indirect striatofugal pathways.

## 1. Introduction

The main pathophysiological feature of Parkinson’s disease (PD) is the loss of nigrostriatal dopamine neurons [1]. L-dopa is the choice of treatment for PD, yet its long-term use causes involuntary and purposeless motor movements known as L-dopa-induced dyskinesia (LID) [2]. They present a behavioral expression of hypersensitive dopamine receptors of striatal medium spiny neurons (MSN) and are a consequence of long-term dopamine depletion in the striatum [3,4] and intermittent stimulation with L-dopa or dopamine receptors agonists [5].

There are contradictory data about the influence of 17-β-estradiol (E2), the main female hormone in the reproductive period [6], on LID. In clinical studies, hormone replacement therapy in menopausal women with developed LID had no effect on LID [7] or has ameliorated only the motor symptoms [8].

In preclinical studies, opposite results could be due to different methodologies of evaluating LID or different sex and animal species used in the studies. Injections of E2 in female *Macaca fascicularis* 1-methyl-4-phenyl-1,2,3,6-tetrahydropyridine (MPTP) model of PD ameliorated LID but had no effect on motor symptoms [9]. In another study that included male 6-hydroxydopamine (6-OHDA) Sprague-Dawley rats, the estradiol receptor antagonist tamoxifen worsened motor complications after L-dopa [10]. They, however, did not assess LID directly but only evaluated the contralateral rotation after L-dopa injections. A rotation test following the application of L-dopa or dopamine agonists has been used to evaluate the hypersensitivity of dopamine receptors of MSN [11]. Because intermittent L-dopa intensifies contralateral rotation [12], it was used as a measure of motor complications. In recent years, a new system of evaluating animal correlates of LID has been established, termed abnormal involuntary movements (AIMs) [13]. In a more recent study, female 6-OHDA rats with removed ovaries developed AIMs later than rats with preserved ovaries which indirectly shows that E2 might worsen LID [14]. Additionally, sex is another important determinant in studies since the effects of E2 are mostly sex specific [15].

The main goal of our study was to define the effect of E2 on LID in female 6-OHDA rats directly with behavioral tests and indirectly with the expression of ΔFosB. ΔFosB is a group of 35–37 kDa isoforms of transcription factor Fos. They are induced after repeated stimuli and remain elevated for a longtime after the cessation of stimuli [16,17]. Its elevated expression has been found in animal models of PD with developed LID [18,19,20] and in the putamen of post mortem PD patients with developed LID [21]. Selective activation of striatal projection neurons that express ΔFosB in animal PD models can induce LID without the application of L-dopa [22].

Additionally, we wanted to explore the effect of E2 on motor impairment using the forepaw adjusted step (FAS) test. The FAS test is used to test animal correlate of bradyhypokinesia [23,24] and as a screening test for nigrostriatal lesions in animal models of PD. The loss of 80% or more of dopamine neurons will cause the animal to make significantly less steps with the forelimb corresponding to the dopamine depleted hemisphere [25].

## 2. Materials and Methods

### 2.1. Animals

Adult female Wistar rats (*Rattus norvegicus*) from the Medical Experimental Centre, Medical Faculty, University of Ljubljana, Slovenia, with an average weight of 230–280 g were used for the experiment. They were housed in polycarbonate cages in a temperature-controlled colony room at 22–24 °C (relative humidity 35–60%) under 12 h light/12 h dark cycle with free access to food and water. To minimize the effect of phytoestrogens in food, a low-phytoestrogen rodent diet was used (Teklad Global 14% Protein Rodent diet 2014, Envigo, Huntingdon, UK).

### 2.2. Experimental Design

At the start of the experiment, all rats were anesthetized using isoflurane and ovariectomized following the procedure described by Zorović et al. (2021) [26]. Two weeks following ovariectomy, 4 µL of 6-hydroxydopamine hydrobromide (6-OHDA, Merck, Burlington, MA, USA) was stereotaxically injected into the right median forebrain bundle as described by Zorović et al. (2021) [26]. The following coordinates were used: 4 mm anteriorly from lambda, 1.3 mm laterally from the midline, and 7.3 mm ventrally from bregma. The animals were assigned to four treatment groups: (1) animals with 17-β-estradiol (E2) implants and daily L-dopa injections (treatment group ‘E2+L-dopa’), (2) animals with empty implants and daily L-dopa injections (treatment group ‘L-dopa’), (3) animals with E2 implants and daily saline injections (treatment group ‘E2+saline’) and (4) animals with empty implants and daily saline injections (treatment group ‘saline’), (Table 1).

**Figure 1:**
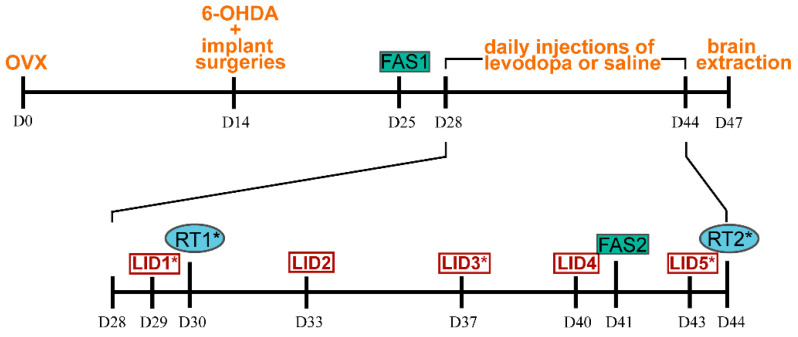
Experimental timeline. Abbreviations: D0–D44: day of the experiment, OVX: ovariectomy, 6-OHDA: 6-hydroxydopamine, FAS: forepaw adjusting step test, LID: L-dopa-induced dyskinesia test, RT: rotation test, *: application of a lower dose of L-dopa prior to LID testing (animals in treatments E2+L-dopa and L-dopa) and rotation testing (all treatment groups).

On the day of the stereotaxic surgery (D14), subcutaneous implants (either empty or filled with E2) were implanted between the scapulae as described by Zorović et al. (2021) [26]. Eleven days later, we performed the first FAS test (Figure 1).

Three days after the first FAS test, we introduced subcutaneous injections of either L-dopa (3,4-dihydroxy-L-phenylalanine, Sigma, Merck KGaA, Darmstadt, Germany, cat. no. D9628; at 4.77 mg kg^−1^) with benserazide hydrochloride (Sigma, Merck KGaA, Darmstadt, Germany, B7283; at 15 mg kg^−1^) or sterile saline at 2 mL per kg of body weight. Injections were administered daily for 16 consecutive days. During this period a second FAS test and two rotational tests (RT) were performed. The rats that received daily L-dopa injections were also tested for LIDs. On the first, third, and fifth LID rating sessions, the rats received a lower dose of L-dopa (3.18 mg kg^−1^) and for the second and fourth sessions they received their regular daily dose. Three days after the last application of L-dopa or saline, the animals were euthanized with CO_2_ and decapitated. Brains were isolated and rapidly frozen on dry ice and kept at −20 °C until they were sectioned in a cryostat (Leica CM1950, Leica Biosystems, Wetzlar, Germany). The brain tissue was cut into 10 μm coronal sections. Sections were mounted to microscope glass slides coated with 0.01% solution of poly-L-lysine (Sigma). Slides with tissue sections were stored at −20 °C. Blood was collected from the chest cavity and/or from the heart and left at room temperature for 20 min, then centrifuged for 10 min at 3000 rpm at room temperature. The serum was aliquoted and kept at −20 °C for further processing.

### 2.3. Behavioural Testing

#### 2.3.1. L-Dopa-Induced Dyskinesias (LIDs)

The assessment of LIDs was carried out during five rating sessions on rats that received daily L-dopa injections. We followed the test procedures and rating protocol of abnormal involuntary movements (AIMs) described by Cenci and Lundblad (2007) [13]. After injection, the rats were placed in separate transparent cages. Over the next three hours, each rat was observed for one minute every twenty minutes (9 measurements per rating session). We assessed the rats for three main types of AIMs: axial AIMs (contralateral trunk and neck flexion), forelimb AIMs (repetitive movements, dystonic movements), and orolingual AIMs (stereotypic jaw movements, tongue protrusions) (Table 2). During each observation period, the amplitude and duration of each AIM were rated from 0–4. The amplitude score for each AIMs subtype was multiplied by its duration score and all three values were summed to give the total AIMs score for each measurement. For each of the five rating sessions, the total AIMs scores were plotted against the timeline. For each animal, the integral of total AIMs scores over time (area under the curve (AUC)) was calculated to give a global AIMs score representing an overall LID assessment combined from 9 observation periods.

#### 2.3.2. Rotation Test (RT)

Rotation testing was done to evaluate the postsynaptic hypersensitivity of the neurons of the direct pathway [11]. Rats were injected subcutaneously with the lower dose of L-dopa with benserazide and immediately placed in 40 cm wide opaque plastic cylindrical arenas. They were attached with a wire harness to the rotation sensor via a flexible tether and observed for two hours. The counting of turns was done by an automated system (Rotameter, Coulbourn Instruments LLC, Whitehall, PA, USA). After two hours, the harnesses were removed, and the animals were returned to their home cages.

#### 2.3.3. Forepaw Adjusted Step Test (FAS Test)

The motor performance FAS test was used to assess the level of bradyhypokinesia. Each rat was held around the torso and one of the forelimbs so that it had to hold its weight with the remaining forelimb. The rat was dragged across the surface at a speed of 10 cm/s, three times in each direction, and the number of steps made by the free forelimb was counted. The process was repeated again with the other forelimb. The number of steps made with the forelimb that presented the dopaminergic deficient hemisphere was expressed as a percentage of steps made with the forelimb that presented the healthy hemisphere. A lower percentage represents a higher level of bradyhypokinesia [23,24,25].

### 2.4. Immunohistochemistry

Chromogenic immunohistochemistry was used to evaluate the loss of dopaminergic neurons in substantia nigra (SN) and the differences in expression of ΔFosB among four treatment groups in lesioned and intact hemispheres. To determine the success of 6-OHDA injections and consequent loss of dopaminergic neurons, we used immunohistochemical labeling of the enzyme tyrosine hydroxylase (TH) on brain slices with SN. Relative optical density was used to assess the amount of immunohistochemically labeled protein in SN and to evaluate the percentage of dopaminergic neuron loss in the lesioned relative to the intact hemisphere.

**Figure 2:**
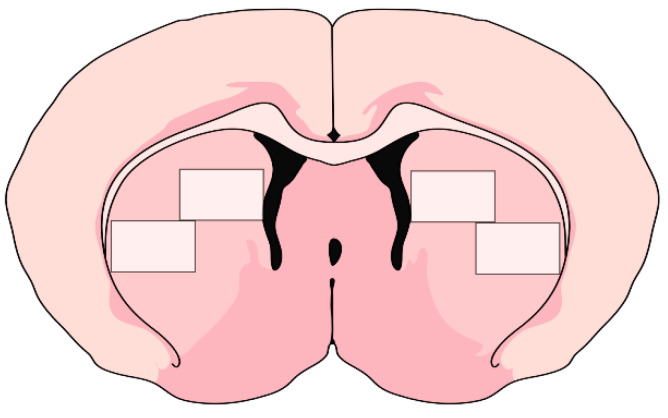
Medial and lateral rostral regions of striatum that were analysed for ΔFosB expression (1 mm anterior to bregma).

To detect the effect of different treatments on plastic changes in the striatal projection neurons, we evaluated the expression of ΔFosB, an activity-dependent transcription factor of the immediate early gene family, which is also one of the molecular markers of LIDs. ΔFosB immunoreactive (ΔFosB-ir) nuclei were counted automatically under 10× magnification in two 2.5 mm^2^ areas in the medial and lateral part of the rostral striatum in the lesioned and the intact hemispheres, 1 mm anterior to bregma (Figure 2) [27].

#### 2.4.1. Chromogenic Immunohistochemical Labelling of TH and ΔFosB

The primary antibodies used were the mouse anti-TH monoclonal antibody (Sigma, Merck KGaA, Darmstadt, Germany, cat. no. T2928) and the mouse anti-FosB (Abcam, Cambridge, UK; cat. no. AB11959). The latter recognizes both FosB and ΔFosB, however, previous studies have shown that FosB degrades well before 24 h post-stimulation, and the vast majority of the immunohistochemical signal that is left after that period is composed of ΔFosB [17]. Since we euthanized the animals and harvested the brains three days after the last application of L-dopa, we can be certain that the immunohistochemical signal shows the expression of ΔFosB. Frozen brain sections were first fixed in cold methanol with 1% H_2_O_2_ for 20 min and then incubated for 1 h at room temperature in a blocking buffer containing 4% normal horse serum, 1% bovine serum albumin (BSA), and 0.4% Triton X-100 in potassium phosphate buffer saline (KPBS: 40 mM K_2_HPO_4_, 10 mM KH_2_PO_4_, 145 mM NaCl; pH = 7.4). After several washes in KPBS, the sections were incubated with either anti-TH or anti-FosB primary antibody (dilution 1:750 and 1:1000, respectively) in KPBS with 1% normal horse serum, 1% BSA, and 0.4% Triton X-100 for 2 h at room temperature. After rinses in KPBS, the sections were incubated with horse anti-mouse biotinylated secondary antibody (Vector Laboratories, Burlingame, CA, USA; cat. no. BA-2001) diluted 1:500 in KPBS with 1% BSA for 90 min at room temperature. After adding the ABC-HRP system (ABC Elite Standard kit, Vector Laboratories, Burlingame, CA, USA; cat. no. PK-6100) to tissue sections, the staining was carried out using 3′3-diaminobenzidine tetrahydrochloride (DAB; Sigma, Merck KGaA, Darmstadt, Germany, cat. no. D5637). The omission of primary antibodies served as a negative control. Sections were dehydrated in ethanol series and cleared in xylene (Carlo Erba reagents SAS, Val de Reuil, France). The sections were mounted with DPX (Sigma, Merck KGaA, Darmstadt, Germany, cat. no. 06522) and coverslipped.

#### 2.4.2. Densitometric Analysis of TH Staining in SN

The microscope slides with tissue sections were placed on a precision illuminator (Northern Light B90, Imaging research Inc., St. Catherine’s, ON, Canada) and the images were captured using MCID Elite 6.0 image analysis software (Imaging Research Inc., St. Catharine’s, ON, Canada), which digitizes the video signal into an 8-bit black-and-white image with 256 shades of gray. Darker areas indicated a higher level of chromogenically tagged protein. For each rat, three adjacent brain slices were imaged. The images of all sections were taken at the same lens settings and lighting conditions. The analysis was performed in Fiji v1.53q. [28]. On each brain slice, we circled equally sized areas comprising SN in both hemispheres, and an equally sized area of the corpus callosum, which represented the background. The software calculated the mean level of grey for each selected area. The optical density (OD) was then calculated as OD = log10 (256/average grey level), with 256 representing the maximum grey value. The background values were subtracted from thus obtained values for SN. The OD values for SN were then expressed as a percentage of the lesioned side relative to the intact side of the brain.

#### 2.4.3. Automated Counting of ΔFosB-ir Nuclei

Brain slices with immunohistochemically labeled ΔFosB protein were imaged at 10× magnification using an inverted microscope (Olympus IX81) coupled to an Olympus DP71 camera (Olympus Life Sciences, Tokyo, Japan). The images were analyzed using Fiji. All images were first converted to 8-bit black-and-white images and the grayscale was adjusted to 256 levels. We then set the parameters to define chromogenically labeled cells for automated counting by Fiji software. We defined the minimum and maximum size of nuclei (to filter out artifacts) and the minimum level of gray to be counted as ΔFosB-ir nuclei. This level was determined by comparing the stained nuclei with controls in which the brain slices were incubated without primary antibodies. Values below this cut-off limit were defined as background on the basis of control slides. We visually checked every labeling of ΔFosB-ir nuclei by Fiji software to ensure there were no missed or mislabeled nuclei.

### 2.5. Serum Levels of 17-β-Estradiol

To determine the concentration of E2 in serum we used Elecsys Estradiol III electrochemiluminescence immunoassay (ECLIA) with the detection limit at 18 pmol/L, and a Cobas e 411 analyzer (both from Roche Diagnostics, Mannheim, Germany).

### 2.6. Statistical Analysis

All statistical analyses were performed with SPSS 27.0 (IBM, Armonk, NY, USA). For the analysis of LID data, we used the nonparametric Mann-Whitney test. We compared the global AIMs scores of treatment groups E2+L-dopa and L-dopa from individual LID rating sessions and total AIMs scores over time during each of the LID rating sessions. We used a one-way ANOVA to analyze differences in LID duration. For the analysis of rotation, FAS tests, and changes in body weight, a three-way mixed ANOVA was used to analyze a possible three-way interaction among between-subjects factors *implant* (E2 or empty) and *injection* (L-dopa or saline) and within-subjects factor *time* (measurement 1 and measurement 2). If no significant three-way interaction was established, a two-way ANOVA was used. Prior to each ANOVA procedure, we used Shapiro-Wilk and Levene’s tests to test for assumptions of normality of distributions and homoscedasticity, respectively. If any of these two assumptions were violated, we either log transformed the data and/or used the Welch statistic or the Brown-Forsythe test. For repeated measures ANOVA we tested sphericity using Mauchly’s sphericity test. In case of violation of sphericity, the Greenhouse-Geisser or Huynh-Feldt corrections were used, depending on the ε value. A two-way ANOVA was used for estimating the effects of factors *implant* or *injection* on the loss of dopaminergic neurons in SN. For the analysis of ΔFosB expression in the medial and lateral rostral striatum, we used a three-way mixed ANOVA with between-subjects factors *implant* (E2 or empty) and *injection* (L-dopa or saline) and within-subjects factor *hemisphere* (lesioned or intact). Additional two-way ANOVA was performed separately for lesioned and intact sides of both striatal regions to test for the effects of factors *implants* and *injection* and their possible interaction. We used a one-way ANOVA with post-hoc Bonferroni correction to analyze the differences in the expression of ΔFosB among the four different treatment groups. The significance level was set at *p* ≤ 0.05. Box plots show the minimum, the maximum, the sample median, and the first and third quartiles.

## 3. Results

### 3.1. Behaviour Analysis

#### 3.1.1. LID Testing

**Figure 3:**
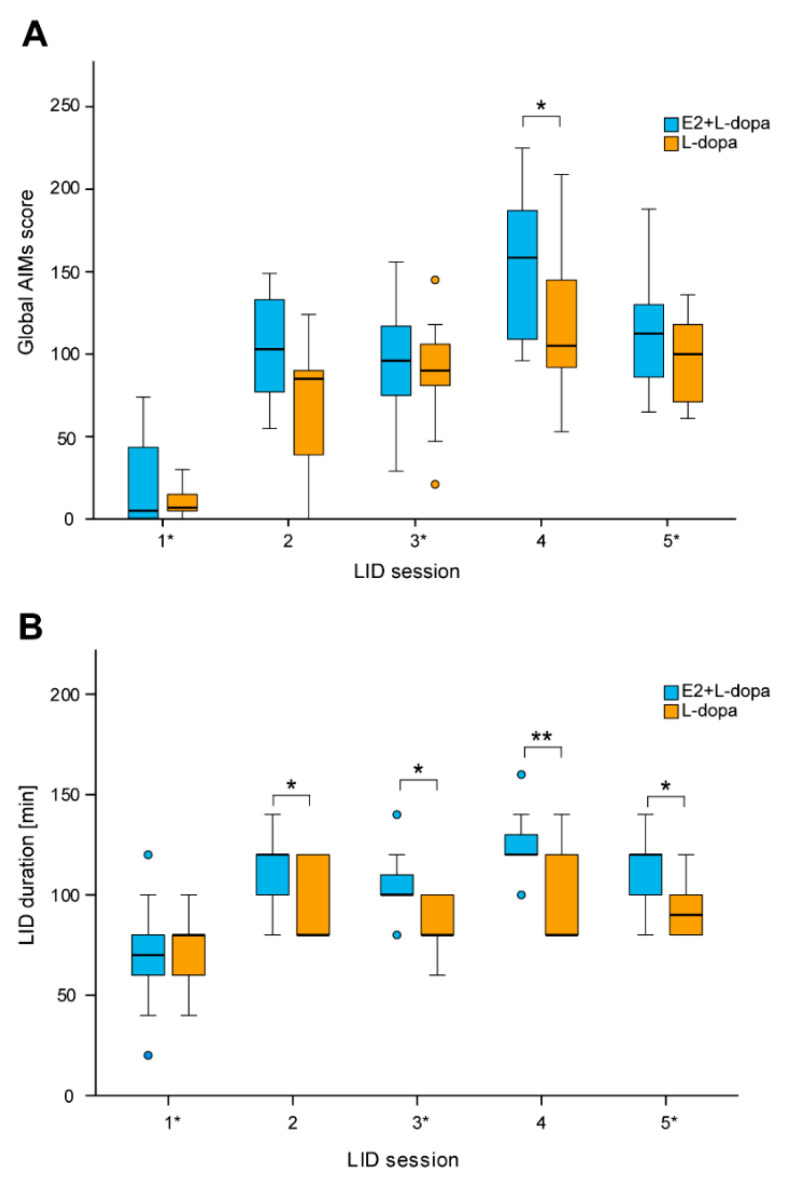
Effect of 17-β-estradiol (E2) on development of L-dopa-induced dyskinesias (LIDs). (**A**) Effect of E2 on global AIMs score in L-dopa treated hemiparkinsonian rats. (**B**) Effect of E2 on duration of LIDs in L-dopa treated hemiparkisonian rats. * *p* ≤ 0.05, ** *p* ≤ 0.01.

The global AIMs score was significantly increased in animals with E2 implants compared to animals with empty implants only in rating session LID4 (*U* = 115, *p* = 0.026) (Figure 3A), while E2 had no significant effect on global AIMs score in sessions 1 (*U* = 67.5, *p* = 0.683), 2 (*U* = 107, *p* = 0.080), 3 (*U* = 85, *p* = 0.605) and 5 (*U* = 92.5, *p* = 0.338). Further analysis showed that the rats that received E2 implants had longer lasting AIMs in all sessions except the first one (session 1: *F*(1,22) = 0.01, *p* = 0.914; session 2: *F*(1,22) = 4.62, *p* = 0.043; session 3: *F*(1,22) = 6.93, *p* = 0.015; session 4: *F*(1,22) = 10.95, *p* = 0.003; session 5: *F*(1,22) = 7.74, *p* = 0.011) (Figure 3B).

**Figure 4:**
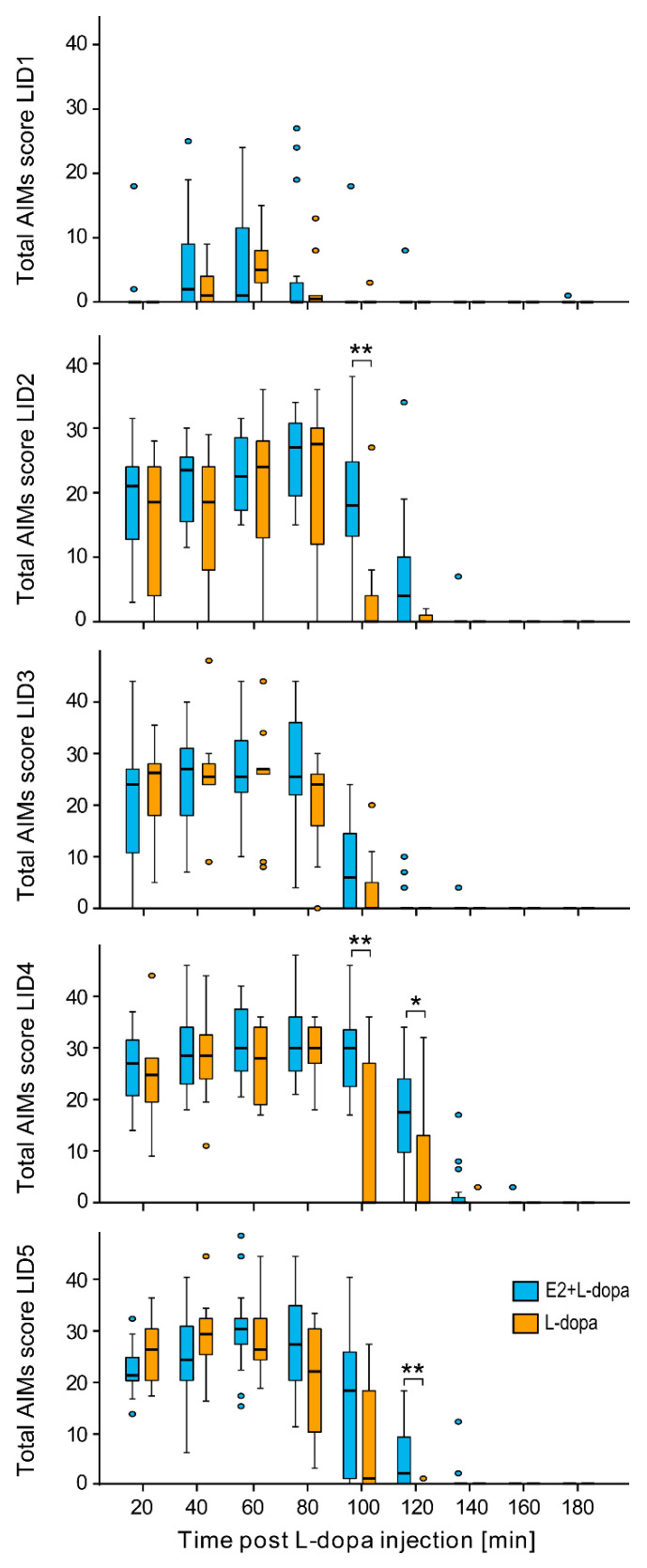
Effect of E2 on the development of AIMs in L-dopa treated hemiparkinsonian rats during individual LID rating sessions. * *p* ≤ 0.05, ** *p* ≤ 0.01.

**Figure 5:**
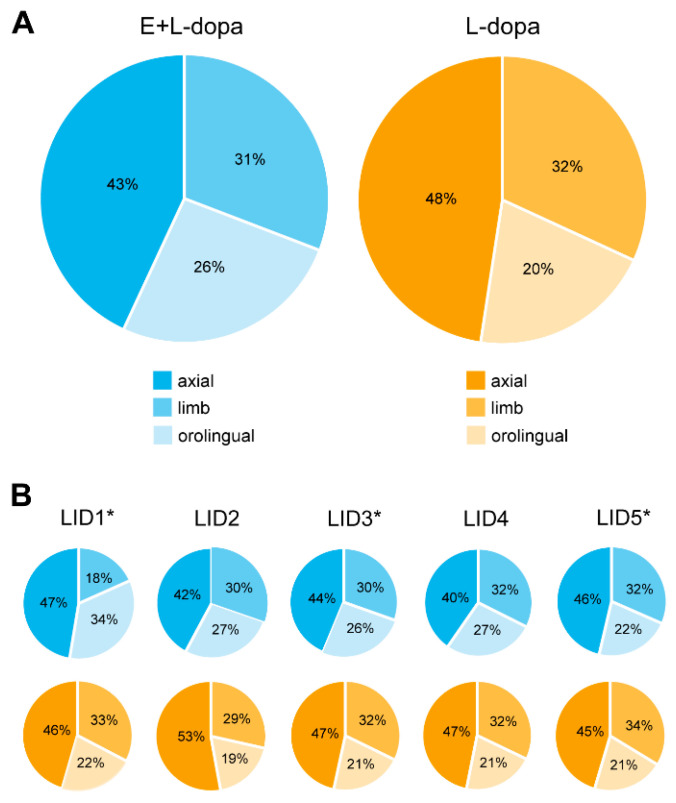
Contributions of different types of AIMs (axial, limb and orolingual) in rating LIDs. (**A**) Contributions of axial, limb and orolingual AIMs to global AIMs score. (**B**) Contributions of axial, limb and orolingual AIMs to total AIMs scores during individual LID rating sessions. *: application of a lower dose of L-dopa prior to LID testing.

Based on these results, we decided to investigate further the AIMs for each session separately by time points. We found that in LID rating sessions LID2 and LID4, E2 significantly increased the total AIMs score in the fifth measurement (100 min post L-dopa injection) (*U* = 120.5, *p* = 0.01 and *U* = 123, *p* = 0.007, respectively) (Figure 4). In LID rating sessions LID4 and LID5, E2 significantly increased the total AIMs score in the sixth measurement (120 min post L-dopa injection) (*U* = 111, *p* = 0.048 and *U* = 126, *p* = 0.004, respectively). The three subtypes of AIMs (axial, limb, orolingual) (Table 2) contributed in different ratios to the total AIMs score. Overall, there was a significantly lower ratio of axial and higher ratio of orolingual AIMs in the treatment group with E2 implants compared to the treatment group with empty implants (*U* = 23, *p* = 0.006 and *U* = 119, *p* = 0.004, respectively) (Figure 5A). The ratio of limb AIMs was not affected by E2. Analysis of single LID rating sessions showed a significant effect of E2 only in sessions 2 and 4. In both, the animals with E2 implants had significantly lower ratios of axial (LID2: *U* = 10, *p* = 0.001; LID4: *U* = 25, *p* = 0.006) and significantly higher ratios of orolingual subtype (LID2: *U* = 111, *p* = 0.009; LID4: *U* = 128, *p* = 0.003) compared to animals with empty implants, whereas the ratio of limb AIMs was not affected (Figure 5B).

#### 3.1.2. Rotation Test

**Figure 6:**
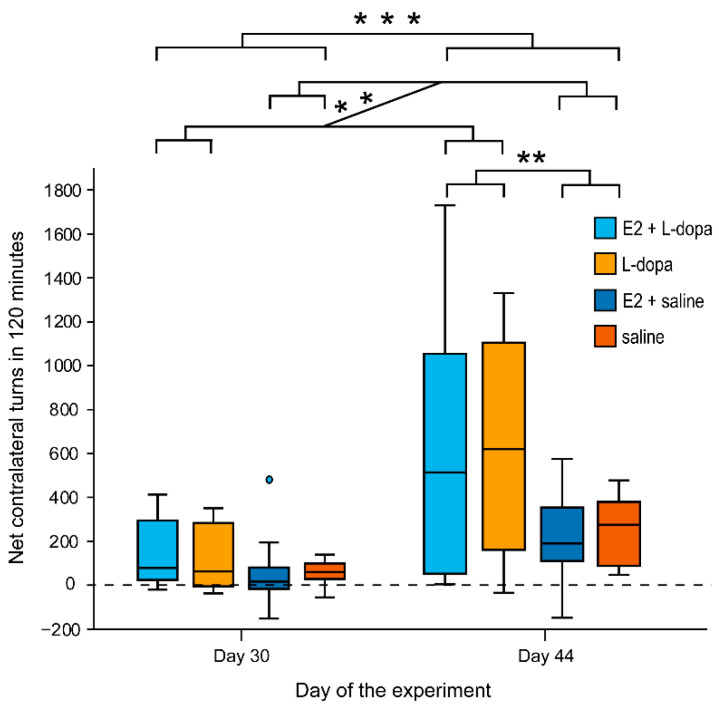
Number of net contralateral turns made by hemiparkinsonian rats in the first (RT1, day 30) and second rotation tests (RT2, day 44). ** *p* ≤ 0.01, *** *p* ≤ 0.001.

**Figure 7:**
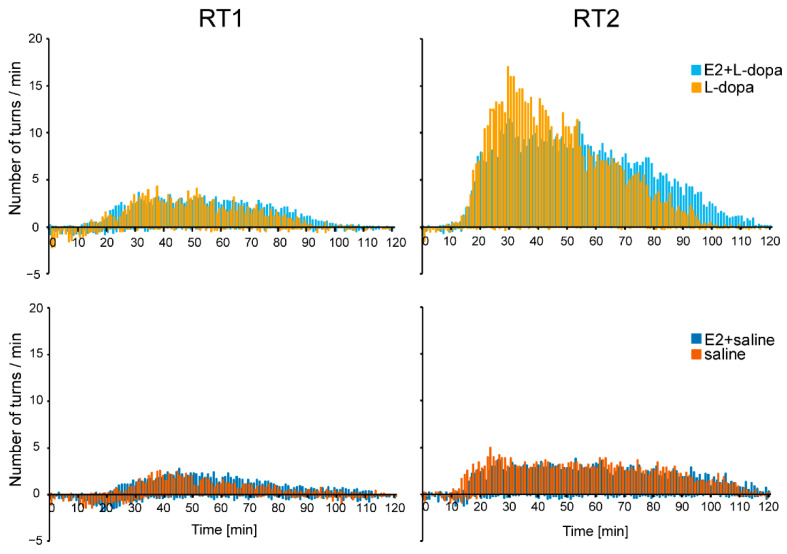
The number of contralateral (positive values) and ipsilateral (negative values) turns per minute during the first (RT1, day 30) and second (RT2, day 44) rotation tests.

E2 had no significant effect on the net contralateral turns on either day 30 or day 44, while factors *injection* and *time* resulted in significant differences in the net contralateral turns (Figure 6). The number of contralateral turns was significantly higher in animals that received daily L-dopa injections vs. animals that received saline injections (both days: *F*(1,38) = 9.61, *p* = 0.004; day 44: *F*(1,38) = 8.981, *p* = 0.005). For all treatment groups, the number of net contralateral turns was significantly higher in the second rotation test (*F*(1,38) = 30.6, *p* ˂ 0.001). Analysis of the number of turns per minute over time similarly shows an increase in the number of contralateral turns in all groups in the second rotation test (Figure 7). Additionally, in the second rotation test, the animals that received daily L-dopa injections (treatment groups E2+L-dopa and L-dopa) showed different temporal dynamics of contralateral rotation depending on the implants. During the first hour, animals with empty implants reached a higher number of turns per minute, whereas, during the second hour, animals with E2 implants scored higher in the number of turns per minute. E2 also resulted in prolonged rotation (ca. 120 min) compared to animals with empty implants (ca. 100 min).

#### 3.1.3. FAS Test

**Figure 8:**
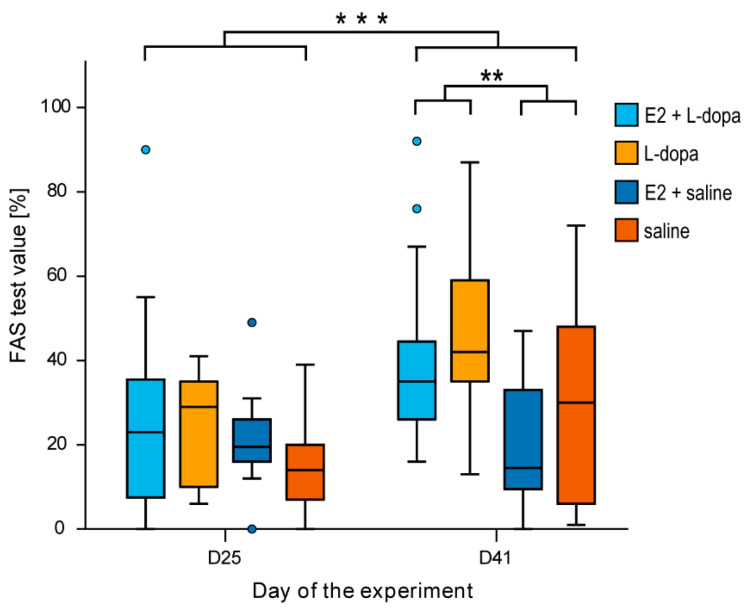
The level of bradyhypokinesia as a result of 6-OHDA lesion was assessed using FAS test. Lower FAS test values indicate higher forelimb motor deficits. ** *p* ≤ 0.01, *** *p* ≤ 0.001.

FAS test was first performed to assess motor deficits in the forelimbs before the onset of daily L-dopa/saline injections and again after almost two weeks of daily L-dopa/saline injections. E2 did not significantly affect the level of bradyhypokinesia on either day 25 or day 44 (Figure 8). However, the development of bradyhypokinesia was significantly affected by factors *time* and *injection*. The motor deficits in the forelimbs were significantly improved in the second assessment (*F*(1,48) = 12.027, *p* = 0.001) and by application of L-dopa (day 44) (*F*(1,48) = 8.397, *p* = 0.006).

### 3.2. Body Weight

**Figure 9:**
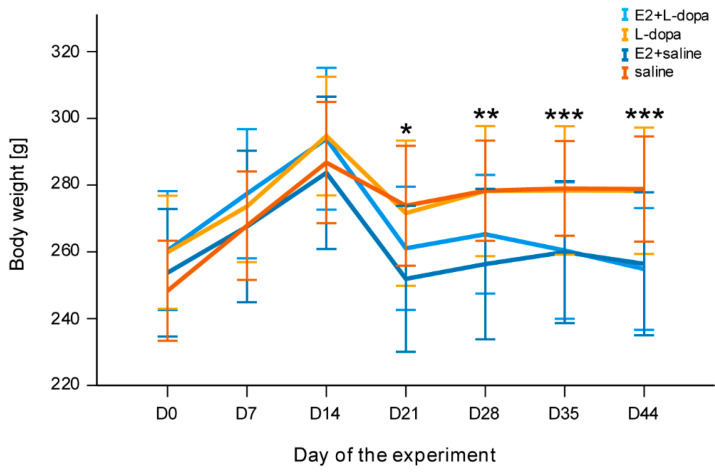
Changes in body weight. The asterisks indicate significant differences between treatment groups based on implants (E2, empty). * *p* ≤ 0.05, ** *p* ≤ 0.01, *** *p* ≤ 0.001.

At the start of the experiment, there were no significant differences in body weight between animals assigned to different treatments. All animals started gaining weight after OVX surgery and losing weight after the 6-OHDA surgery (Figure 9). Until the end of the experiment, weight loss was significantly affected by factors *time* (*F*(6,276) = 87.942, *p* ˂ 0.001), *time* × *implants* (*F*(6, 288) = 24.718, *p* ˂ 0.001) and *time* × *injections* (*F*(6,288) = 4.014, *p* = 0.024). One week after the stereotaxic injection of 6-OHDA, a decrease in body weight was significantly higher in animals with E2 implants compared to animals with empty implants (*F*(1,48) = 6.982, *p* = 0.011). The changes in weight gain in the following weeks were significantly affected by E2 (D28: (*F*(1,48) = 9.457, *p* = 0.003), D35: (*F*(1, 48) = 11.876, *p* = 0.001, D44: (*F*(1,48) = 18.836, *p* ˂ 0.001), but not by the presence or absence of L-dopa. Animals with empty implants gained more weight than animals with E2 implants.

### 3.3. Serum E2 Levels

As expected, the level of E2 was significantly higher in rats with E2 implants (M = 261 ng/L, SD = 72.4) compared to rats with empty implants (M = 9.39 ng/L, SD = 72.8; *F*(1,47) = 140, *p* < 0.001).

### 3.4. Immunohistochemical Analysis

#### 3.4.1. Loss of Dopaminergic Neurons in SN

**Figure 10:**
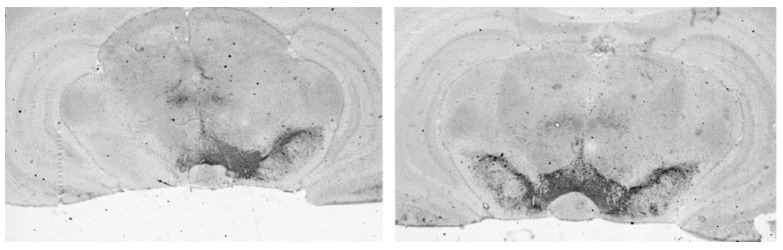
Loss of dopaminergic neurons in 6-OHDA lesioned rats. Representative sections showing TH positive neurons in SN in a rat with a successful unilateral 6-OHDA lesion (**left**) and an unsuccessful unilateral 6-OHDA lesion (**right**).

The overall percentage of dopaminergic neuron loss on the lesioned side was 89.4 ± 2.2% (Figure 10). There were no statistically significant differences in the extent of loss of dopaminergic neurons on the lesioned side of the brain among the four treatment groups (Table 3).

#### 3.4.2. Expression of ΔFosB

**Figure 11:**
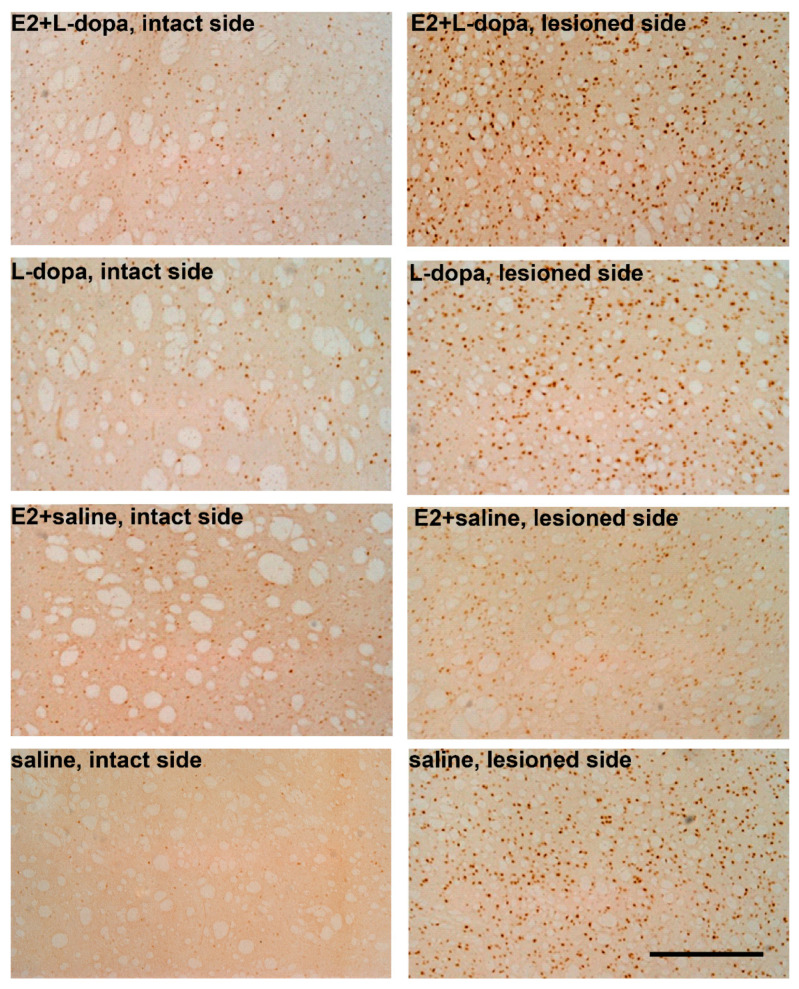
Photomicrographs of ΔFosB immunoreactive nuclei (ΔFosB-ir; dark brown spots) in coronal sections of lateral rostral striatum from animals in treatment groups E2+L-dopa, L-dopa, E2+saline and saline. (**Left**)—intact hemisphere; (**right**)—lesioned hemisphere. Scale: 500 µm.

**Figure 12:**
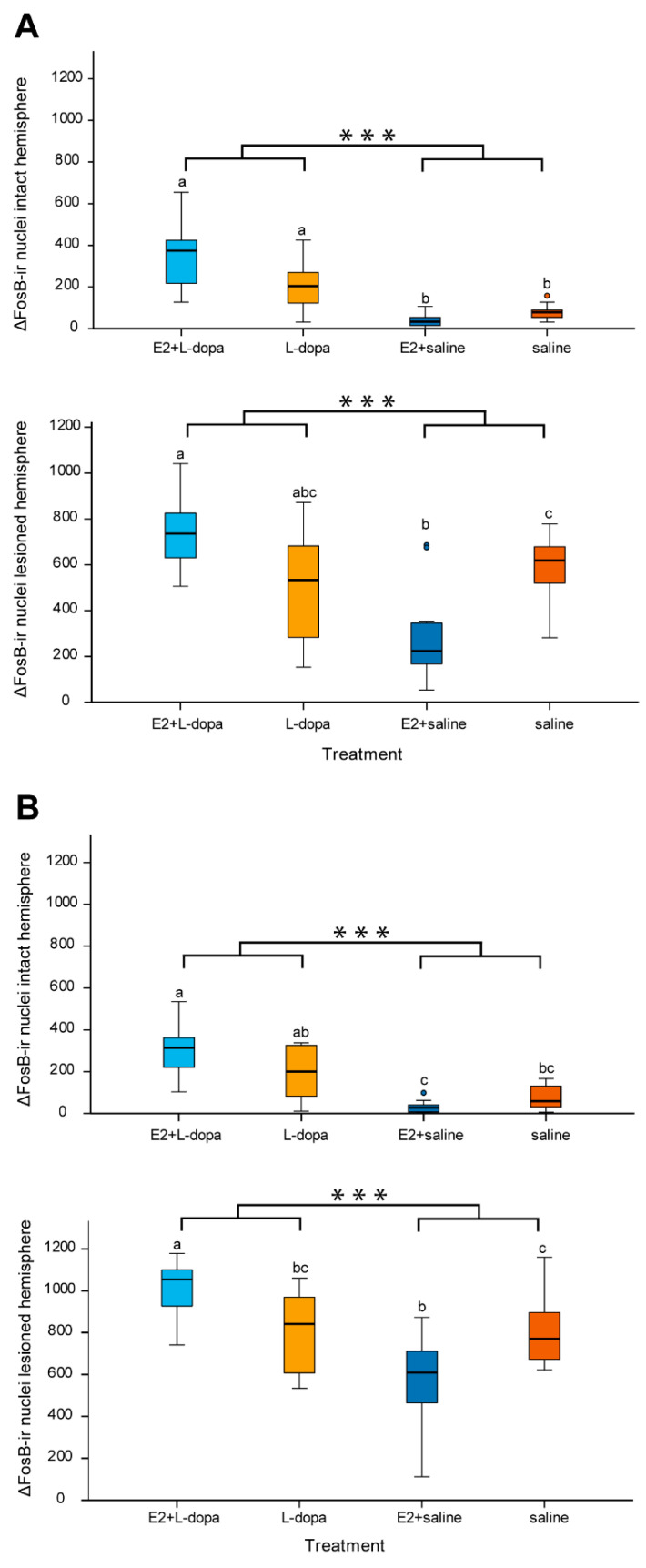
Expression of ΔFosB in the intact and lesioned hemispheres of the medial (**A**) and lateral (**B**) rostral striatum. The asterisks indicate statistically significant differences between treatment groups that received daily L-dopa injections (E2+L-dopa and L-dopa) and those that received daily saline injections (E2+saline and saline) (*** *p* ≤ 0.001). Different letters above box plots indicate significant differences between treatments (*p* < 0.05). Means with no letters in common are significantly different, while means with at least one common letter are not significantly different.

In both medial and lateral area of rostral striatum, a three-way mixed ANOVA showed statistically significant effects of factors *hemisphere* (medial striatum: *F*(1,46) = 377,589, *p* < 0.001; lateral striatum: *F*(1,46) = 994.433, *p* < 0.001) and *injection* (medial striatum: *F*(1,46) = 12.698, *p* = 0.001; lateral striatum: *F*(1,45) = 19.199, *p* < 0.001) on the expression of ΔFosB, as well as two-way (*implant × injection*: medial striatum: *F*(1,46) = 24.483, *p* < 0.001; lateral striatum: *F*(1,46) = 19,265, *p* < 0.001, and *hemisphere × injection*: medial striatum: *F*(1,46) = 12,568, *p* = 0.001; lateral striatum: *F*(1,46) = 19.027, *p* < 0.001) and three-way interactions between different factors (*hemisphere × implant × injection*: medial striatum: *F*(1,46) = 24.472, *p* < 0.001; lateral striatum: *F*(1,46) = 19,239, *p* < 0.001) (Figure 11). A two-way ANOVA was performed separately for lesioned and intact sides of both striatal regions to test the effects of factors *implants* and *injection* and their possible interaction. E2 alone had no significant effect on the expression of ΔFosB in either lesioned or intact hemispheres, however, there was significant interaction between factors *implants* and *injection* in both hemispheres and regions (intact medial striatum: *F*(1,46) = 13.825, *p* < 0.001; lesioned medial striatum: *F*(1,46) = 24.980, *p* < 0.001; intact lateral striatum: *F*(1,46) = 9.497, *p* = 0.003; lesioned lateral striatum: *F*(1,46) = 19.252, *p* < 0.001). L-dopa had a significant effect on ΔFosB in medial and lateral striatum of both hemispheres (intact medial striatum: *F*(1,46) = 80.494, *p* < 0.001; lesioned medial striatum: *F*(1,46) = 12.782, *p* = 0.001; intact lateral striatum: *F*(1,46) = 44.480, *p* < 0.001; lesioned lateral striatum: *F*(1,46) = 19.113, *p* < 0.001) (Figure 12). Additional one-way ANOVA was used to analyse the differences in the expression of ΔFosB among the four different treatment groups. This analysis was performed separately for each region and each hemisphere. In animals that received only saline injections, E2 always resulted in a lower expression of ΔFosB compared to animals with empty implants, but the difference was significant only in the lesioned hemispheres (*p* = 0.04). Inversely, in animals that received L-dopa, E2 caused an increase in ΔFosB expression, which was, however, statistically significant only in the lateral striatum of the lesioned hemisphere.

## 4. Discussion

Our study showed that in female 6-OHDA rats E2 potentiated the effect of intermittent L-dopa injections, displayed worse AIMs and elevated expression of ΔFosB. E2 also changed the temporal dynamics of contralateral rotation. On the other hand, it did not influence motor impairment.

E2 had no effect on the survival of nigrostriatal neurons although some studies showed a neuroprotective role of E2 [29]. This might be due to the fact that in those studies E2 was able to protect dopamine neurons only in case of partial lesions [30] or if the animals received E2 at least 24 h before 6-OHDA injections [31]. Because previous studies showed that the extent of dopamine loss correlates with the severity of AIMs [18] and the expression of ΔFosB [32] in the 6-OHDA model of PD, the equal levels of neuronal loss allowed us to objectively compare different groups (Table 3).

### 4.1. AIMs

We found that E2 significantly worsened AIMs (Figure 3), which has also been shown in a recent study where higher E2 status in female rats correlated with worse AIMs; 6-OHDA female rats with intact gonads developed AIMs sooner than ovariectomized rats [14]. In our study, E2 prolonged AIMs but had no significant impact on their amplitude. Analysis of individual testing sessions showed significantly higher AIM scores in rats with E2 implants in later time points when AIMs were abating (Figure 4). This was seen in the second and fourth sessions, when a higher dose of L-dopa was used, and in the fifth session when we observed maximum amplitude of AIMs. One reason why E2 could not worsen the amplitude of AIMs is that in those testing sessions majority of rats already achieved the maximal amplitude of AIMs according to the AIM rating system.

Another possibility could be that while E2 increased orolingual dyskinesias it decreased axial dyskinesias (Figure 5A). Although we analyzed the percentage of loss of nigrostriatal neurons in the SNpc and found no difference between the groups, we did not analyze the depletion of dopamine fibers in the striatum. Studies showed that the striatum is somatotopically organized [33], and an uneven survival of dopamine fibers between groups could explain differential impact on different subtypes of AIMs [34]. For example, Andersson et al. (1999) showed that dopamine denervation of the dorsolateral striatum was associated with orolingual AIMs [19].

### 4.2. Rotation Test

Rats that received daily L-dopa injections performed more contralateral turns compared to rats that received saline (Figure 6). This was expected since previous studies showed that intermittent L-dopa increases contralateral rotation by inducing hypersensitivity of the D1 dopamine receptors of the direct medium spiny neurons (dMSN) [12,35]. While E2 had no significant effect on the total number of contralateral turns, it changed the temporal dynamics of rotation in this group. As seen in Figure 7, it decreased the amplitude in the first hour and, similarly as seen in AIMs, prolonged the behavior in the second hour of the rotation test. We observed that in the first hour after receiving L-dopa, severe AIMs abruptly interfered with rotation (Figure 7). This phenomenon was already described by Ungerstedt [11], yet studies rarely report this effect. An almost direct comparison of the timeline of AIMs and rotation is seen in the study by Ryan et al. (2018). Following L-dopa injections, higher turning scores are observed in animals with E2 implants. Shortly after, rotation decreases and is replaced by higher AIM scores and later rotation again replaces AIMs [36]. Higher intensity of AIMs in rats with E2 implants (see above in the discussion of AIMs) could have interfered with the rotation by lowering the number of turns at the time of the highest AIMs. It is also important for future studies to report the rotational temporal profile and not only the net turns.

At the time of the maximal amplitude of AIMs, the axial dyskinesia is described as bending the torso for more than 90 degrees contralateral and losing balance. These movements imitate turning and could wrongfully be accounted for contralateral turns by the automatic rotometer. Because axial dyskinesia was the prevailing subtype of dyskinesia in rats with empty implants, it is possible that a part of these AIMs have accounted for contralateral turns, meaning that rats with empty implants might not have performed more turns compared to the rats with E2 implants.

Another reason for the significant influence of E2 on AIMs but not on contralateral rotation might be due to different underlying mechanisms. While hypersensitivity of the D1 receptors of dMSN has been shown to be the probable culprit for AIMs [22,36], different subpopulations of the dMSN were discovered [22,37]. In a previous study, these subpopulations of dMSN were shown to be selectively responsible for the AIMs or for the rotation [36]. How E2 exerts its effect on different subpopulations of the dMSN is beyond the scope of our study and future studies are needed need to address this possibility.

Rats that received saline also performed more contralateral turns in the second compared to the first testing (Figure 6 and Figure 7). This could be due to motor learning, a second L-dopa dose (contralateral rotation intensifies with an increasing total dose of L-dopa) or because of additional nigrostriatal cell death after two weeks (rotation after L-dopa is dependent on the lesion size).

### 4.3. Expression of ΔFosB

In the group of rats that received daily saline, the expression of ΔFosB was higher in the dopamine denervated striatum than in the intact hemisphere (Figure 12), which has also been shown by other studies [16,38]. We believe that this expression mainly happens in the indirect medium spiny neurons (iMSN) since the loss of dopamine results in their disinhibition. At the same time, due to a shortage of dopamine, dMSN are not activated. In the study by Cenci et al. (1999), there was almost no overlap between the expression of FosB and dynorphin (levels rise after activation of dMSN) in the striatum of 6-OHDA rats which could indirectly confirm that the majority of FosB was expressed in the iMSN [20].

Additionally, there was a lower expression of ΔFosB in the striatum of rats with E2 implants. If our assumption is correct, then E2 was able to at least partially lower/inhibit the activity of iMSN in the denervated striatum. This could result in higher thalamocortical input and could therefore improve the motor symptoms of PD. This could explain some clinical studies that associate higher E2 hormonal status (longer fertility period, higher number of children) with a later onset of motor symptoms in PD [39,40,41]. However, we did not detect any effect of E2 on bradyhypokinesia in our study (Figure 8).

There was also a higher expression of ΔFosB in the dopamine denervated striatum of rats that received daily L-dopa injections, which has also been shown previously [16,20]. More importantly, in rats that received daily L-dopa injections, E2 increased the level of ΔFosB expression (Figure 12). Because intermittent L-dopa hypersensitizes the D1 dopamine receptors of dMSN, we assume that this expression is localized in dMSN. Several studies showed the expression of ΔFosB in the dMSN in 6-OHDA models that received daily L-dopa injections [18,19]. Additionally, selective activation of dMSN in 6-OHDA animal models of PD was able to induce AIMs [42]. Higher activation of dMSN due to E2 could result in an even higher thalamocortical output, resulting in more severe LID. This was corroborated by our behavioral tests which indeed showed worse AIMs in rats that received E2 implants.

The effect of E2 on ΔFosB expression only seems contradictory at first. We assume that E2 does not influence the dMSN and iMSN selectively but only amplifies the physiological effect of dopamine. The different effect on the direct and indirect pathway is the result of different treatment (saline vs. L-dopa). In the group that received saline, E2 lowered the activity of iMSN, while it exaggerated the already hyperactivated dMSN in the group that received L-dopa.

### 4.4. The FAS Test

In the second testing session, L-dopa treated rats performed better than saline treated rats, while no effect of E2 on the FAS test was detected (Figure 8). We propose that this is due to the different pharmacodynamics of L-dopa (acute injections before testing of rotation and AIMs) and dopamine in the remaining nigrostriatal neurons. L-dopa is converted to dopamine in the remaining nigrostriatal neurons to compensate for the dopamine loss [43]. This could represent a »basal dopamine level« and explain why rats with intermittent L-dopa performed better than rats with the saline application, even in the absence of an acute L-dopa injection. However, L-dopa also causes contralateral rotation which is suggestive of a postsynaptic mechanism of action, such as of D1 receptor agonists [44]. Furthermore, experiments showed that co- injections of L-dopa and D1 antagonists block contralateral rotation [45]. We assume that a possible agonist effect of L-dopa on D1 receptors is of quicker action than that of conversion to dopamine and that it prevails in the tests of AIMs and contralateral rotation. Because E2 amplifies the already hypersensitive D1 receptors, E2 exerts its effect only when an acute injection of L-dopa is used.

On the other hand, the FAS test is a test of motor impairment and therefore assessed without an acute L-dopa injection. We presume that because of this, the conversion of L-dopa to dopamine mechanism prevails, thus securing a »basal dopamine level«. Additionally, a previous study showed that in the dopamine denervated striatum of 6-OHDA rats, locomotion did not affect the firing rate of dMSN but only decreased the firing rate of iMSN [36].

### 4.5. Heterogeneity of Data

Apart from defining the effect of E2 on LID, our aim was to illustrate the complexity and crudeness of behavioral assessments. We believe that the most appropriate method of presenting our data is the box plot graphs, which illustrate the wide spread of the behavioral data (especially of LID) and a narrower spread of ΔFosB expression data. Previously described potential mechanisms influencing behavior might lead to very diverse behavior, and although gross differences are visible from the graphs, subtle changes are harder to detect. This data dispersion might hinder the acknowledgment of subtle changes as statistically significant, which could therefore be overlooked.

### 4.6. Weight Loss

Although weight monitoring was not the primary aim of our study, we found it important to present these results as a relevant factor in future studies and as an indicator of animal wellbeing.

Many studies have reported weight loss in patients with PD [46,47,48]. This is what we also observed in our study, when rats lost weight after a 6-OHDA injection (Figure 9). It could be a consequence of lower calorie intake because of loss of appetite due to smell loss [49], obstipation [50], or depression [46]. On the other hand, higher energy output due to muscle rigidity could also lead to weight loss [51].

We were also interested in rats’ weight because therapy with L-dopa is associated with lower weight [46]. Additionally, LID could present an additional energy output. We have not found any influence of L-dopa on weight.

Weight gain after OVX and weight loss after additional E2 are known effects [52] that are being used in the research of postmenopausal experiment models [53] and studies of the effect of E2 on the pathophysiology of diseases [54,55].

## 5. Conclusions

Our study showed that E2 significantly prolonged AIMs and increased the expression of ΔFosB, possibly by potentiating the L-dopa-induced hypersensitivity of D1 receptors of MSN. Although we found no significant effect of E2 on net contralateral rotations and motor impairment, E2 did change the temporal dynamics of rotation. Similarly, as seen in AIMs, the observed behavior was prolonged. Therefore, the net turns of contralateral rotations after an acute L-dopa injection should be used only as a crude estimate of LID development in animal models of PD. Further studies are necessary to investigate the underlying mechanism by which E2 exerts its effects on LID.

## Figures and Tables

**Table 1:** Treatment groups.

Treatment Group	Implants	Daily Injections ^1^	n
E2+L-dopa	E2	L-dopa	15
L-dopa	Empty	L-dopa	10
E2+saline	E2	Saline	12
Saline	Empty	Saline	13

^1^ Daily injections were applied from day 28 to day 44 of the protocol (see Figure 1). Abbreviations: E2: 17-β-estradiol.

**Table 2:** LID assessment rating system.

		Axial AIMs	Limb AIMs	Orolingual AIMs
**amplitude**	1	Sustained deviation of head and neck, at ~30° angle.	Tiny movements of the paw around the snout.	Twitching of facial muscles, small masticatory movements without jaw opening.
	2	Sustained deviation of head and neck, angle ≤ 60°.	Movements of the whole limb up and down from the floor to the snout.	Twitching of facial muscles, noticeable masticatory movements occasional jaw opening.
	3	Sustained twisting of the head, neck, and upper trunk at an angle > 60° but ≤90°.	Large displacement of the whole limb with visible contraction of shoulder muscles, digital extension.	Movements with broad involvement of facial muscles and masticatory muscles, frequent jaw opening, occasional tongue protrusion.
	4	Sustained twisting of the head, neck, and trunk at maximal amplitude (angle > 90°), causing the rat to lose balance (from a bipedal position).	Dystonic posture with elbow extension, digital flexion into fists.	All the above muscle categories are involved to the maximal degree.
**duration**	0	No dyskinesia.		
	1	Less than 30 s.		
	2	More than 30 s.		
	3	Continuous dyskinesia during the entire observation time, but is suppressible by external stimuli (hand clapping).		
	4	Continuous dyskinesia during the entire observation time that is not suppressible by external stimuli.		

Adapted from Cenci and Lundblad, 2017 [13].

**Table 3:** Loss of dopaminergic neurons in substantia nigra pars compacta (SNpc) on the lesioned side.

Treatment Group	Percentage of Neuron Loss in SN ($\bar{x}$ ± SD)	n
E2+L-dopa	88.7 ± 5.8	15
L-dopa	89.9 ± 6.2	10
E2+saline	91.7 ± 6.6	12
Saline	87.6 ± 6.3	13

## Data Availability

The data presented in this study are openly available in Mendeley Data repository, doi:10.17632/kvptfg6chy.3.
